# Seeking a Fast Screening Method of the Varietal Origin of Olive Oil: The Usefulness of an NMR-Based Approach

**DOI:** 10.3390/foods10020399

**Published:** 2021-02-11

**Authors:** Maria João Cabrita, Arona Pires, Anthony J. Burke, Raquel Garcia

**Affiliations:** 1 MED—Mediterranean Institute for Agriculture, Environment and Development, Departamento de Fitotecnia, Escola de Ciências e Tecnologia, Universidade de Évora, Pólo da Mitra, Ap. 94, 7006-554 Évora, Portugal; raquelg@uevora.pt; 2 Departamento de Química, Escola de Ciências e Tecnologia, Universidade de Évora, Rua Romão Ramalho, 59, 7000-671 Évora, Portugal; arona_pires@hotmail.com (A.P.); ajb@uevora.pt (A.J.B.); 3 Escola Superior Agrária, Instituto Politécnico de Coimbra, Bencanta, 3045-601 Coimbra, Portugal; 4 LAQV-REQUIMTE, Universidade de Évora, Rua Romão Ramalho, 59, 7000-671 Évora, Portugal

**Keywords:** olive oil, varietal origin, ^1^H NMR, ^13^C NMR DEPT 45

## Abstract

This work encompasses the use of 1D multinuclear NMR spectroscopy, namely, ^1^H NMR and ^13^C NMR DEPT 45, combined with a multivariate statistical analysis to characterize olive oils produced from nine different varieties: Galega Vulgar, Cobrançosa, Cordovil de Serpa, Blanqueta, Madural, Verdeal Alentejana, Arbequina, Picual and Carrasquenha. Thus, the suitability of an NMR-based spectroscopic tool to discriminate olive oils according to their varietal origin is addressed. The results obtained show that the model based on ^13^C NMR DEPT 45 data has a stronger performance than the model based on ^1^H NMR data, proving to be promising in the discrimination of the olive oils under study based on their varietal origin, being particularly relevant for olive oils of the Galega Vulgar variety.

## 1. Introduction

In the last 15 years, the Alentejo region, located in the south of Portugal, has gained 50 thousand hectares of olive orchards and is nowadays responsible for approximately 70% of Portuguese olive oil (OO) production. Typical varieties in this region include Galega Vulgar, Cobrançosa, Cordovil de Serpa and Verdeal Alentejana. However, in recent orchards, other varieties, including foreign ones, can be found. Due to the socio-economic relevance of Alentejo’s olive orchards, the valorization of varietal olive oils from the most important traditional cultivars is mandatory, of paramount relevance being the study of olive oils produced from Galega Vulgar, one of the oldest varieties.

Few studies related to olive oils produced in Portugal using the typical olive varieties are described in the literature, mainly based on the use of chromatographic and spectroscopic techniques and chemometric tools [1,2,3,4,5,6]. Peres and coworkers [1] used artificial neural networks to achieve a chemometric classification of olive cultivars from Cobrançosa, Cordovil, Madural, Negrinha de Freixo, Santulhana and Verdeal Transmontana from the Trás-os-Montes region (northeast of Portugal). The characterization of aroma compounds in Portuguese extra virgin olive oils extracted from two olive cultivars—Galega Vulgar and Cobrançosa—grown in the Beira Baixa region of central Portugal was studied by Peres and coworkers [7]. Other studies reported by Vaz Freire and collaborators used comprehensive gas chromatography in tandem with time-of-flight mass spectrometry (GC × GC/ToFMS) to analyze the aroma compounds of olive oils from Galega, Carrasquenha and Cobrançosa from Alentejo [2]. More recently, an assessment of the volatile profile of varietal olive oils of nine cultivars from Alentejo was performed using an approach based on revisited 3D van Krevelen diagrams [3]. Other research work has applied short-wavelength Raman spectroscopy and Fourier transform infrared spectroscopy (FTIR) for the discrimination and classification of Cobrançosa, Galega Vulgar and Picual olive oils [4,5]. More recently, 82 monovarietal extra virgin olive oil (EVOO) samples from nine different cultivars in the Alentejo region were used to assess the fatty acid component by Fourier-transform near-infrared spectroscopy (FT-NIRS) [6].

In the last few years, nuclear magnetic resonance (NMR) spectroscopy has emerged as a promising tool in studies concerning the authenticity, quality and geographical origin of olive oils [7,8,9]. In particular, NMR has proven to be an efficient technique in the fatty acid characterization of olive oil samples, enabling the assessment of fatty acid profiles as well as the ratio between different acyl groups, playing a crucial role in the study of the quality of these edible oils, the screening of the oxidation and deterioration process, the assessment of purity and authenticity and adulteration practices [10]. Indeed, NMR is a robust quantitative analytical technique used to obtain “high-throughput” spectroscopic and structural information on a wide range of molecular compounds [11,12,13,14]. The main advantages of NMR analysis are that (i) it can be used without using specific standards, (ii) it provides structural information, (iii) it is a non-destructive method, (iii) one can analyze simultaneously various components in a mixture without pre-isolation and (4) it requires a relatively short analysis time compared to other conventional analytical techniques (such as GC and HPLC), and the preparation of the sample is very simple and rapid [15]. Due to the higher sensitivity and lower relaxation times of proton nuclei, ^1^H NMR spectroscopy is the most widely used method for most NMR applications [16]. Several authors have used this spectroscopic methodology, combined with different statistical tools, to achieve olive oil classification according to cultivars, particularly using ^1^H NMR data [17,18,19,20]. The principal limitation of ^1^H NMR spectroscopy is the complex nature of the ^1^H NMR spectrum, which contains several overlapping peaks due to the presence of different multiplet patterns that arise from spin coupling of the different protons, which are condensed into a very narrow spectral window (~15 ppm). Another limitation of ^1^H NMR spectroscopy is that it is not possible to see the positional distribution of the fatty acids in the glycerol backbone [15].

Despite the low relative abundance of the ^13^C isotope, ^13^C NMR is another very powerful NMR technique that allows the assignment of different carbons within the organic molecule. In particular, the use of ^13^C NMR to discriminate the variety of extra virgin olive oils using multivariate statistical analysis has already proven to be a useful tool [16,21]. In order to simplify the analysis of the spectra, the carbons are decoupled from the protons using a technique known as broadband decoupling [22], which furnishes a spectrum consisting of straight lines of varying intensities. Carbon peak assignments are effectively made using the technique of distortionless enhancement by polarization transfer (DEPT), which permits useful spectral editing of the spectra, distinguishing between the tertiary, secondary, primary and quaternary carbons [22]. The main advantage of this technique, besides distinguishing among the different carbons, is that the sensitivity (good ratio signal/noise (S/N)) of the experiment is improved [22]. The advantages of the DEPT experiment in the analysis of olive oil samples have been convincingly addressed by Vlahov and coworkers [23] and ensure that the resonance intensity measurements are accurate and precise, minimizing differences in the olive oil constituents due to measurement errors and pin-pointing the oil variability based on different varietal and/or geographic origins [24].

Moreover, in our previous publication, we showed the power and potential of DEPT editing techniques for assessing variations in the compositions of different olive oils [15]. Variation in the peak heights of the carbons present in the triacylglycerol fraction was used as the means to determine variability within olive oil samples. In fact, in the DEPT 45 experiment, all the peaks from primary to tertiary carbons were observed in the spectrum, which is particularly advantageous for our purposes in this study.

Thus, aiming to assess the varietal origin of olive oils produced from nine different varieties (Galega Vulgar, Cobrançosa, Cordovil de Serpa, Blanqueta, Madural, Verdeal Alentejana, Arbequina, Picual and Carrasquenha), an NMR-based approach was applied in this study, and its usefulness in the fast screening of OO varietal origin discrimination was investigated. Since the ^1^H NMR spectrum allows one to have information about major and minor olive oil components, whereas ^13^C NMR can provide valuable information about the acyl distribution and the acyl positional distribution of glycerol tri-esters of different oils, in this work, two experiments were applied due to their complementary data. Concretely, this study uses the resonance intensities of triacylglycerols obtained by ^1^H NMR spectroscopy and a ^13^C NMR DEPT45 pulse sequence to classify olive oils according to varietal origin by applying a linear discriminant analysis (LDA).

## 2. Materials and Methods

### 2.1. Olive Oil Sampling

This study was performed on 125 varietal olive oils (OO) from two consecutive years and from nine different varieties of *Olea europaea* L.: Galega Vulgar (4 OO from 2016 and 22 OO from 2017), Cobrançosa (7 OO from 2016 and 12 OO from 2017), Arbequina (5 OO from 2016 and 12 OO from 2017), Cordovil de Serpa (6 OO from 2016 and 9 OO from 2017), Blanqueta (9 OO from 2016 and 7 OO from 2017), Picual (5 OO from 2016 and 6 OO from 2017), Carrasquenha (2 OO from 2016 and 7 OO from 2017), Verdeal Alentejana (2 OO from 2016 and 5 OO from 2017) and Madural (2 OO from 2016 and 3 OO from 2017). Olives were collected from georeferenced production sites at approximately the same ripening stage over the period of October and November of 2016 and 2017, and olive oils were obtained in commercial milling facilities or produced in an Abencor system (Seville, Spain) from fresh olives (approximately 5 kg of each olive variety). Samples were kept cool and in dark glass bottles until analysis, which was performed within the first two months of bottling.

### 2.2. NMR Experiments and Sample Preparation

The triacylglycerol fraction (TAG) was analyzed using 1D multinuclear NMR spectroscopy (^1^H NMR, ^13^C NMR DEPT 45). The NMR experiments were conducted on a Bruker Advance III 400 MHz spectrometer (Madrid, Spain), equipped with a wide-band (BBO) observation probe at a temperature of 303K. The generated files were handled by Topspin software 3.2 pl 6.

For the olive oil sample analysis, the following procedure was used. In a 5mm diameter NMR tube, 100 μL of olive oil and 10 μL of mesitylene (internal standard) were dissolved in 500 μL of deuterated chloroform. Each sample was analyzed by means of the two 1D-NMR experiments that encompass ^1^H NMR (Figure S1) and ^13^C NMR DEPT 45 (Figure S2). The pulse sequences used in these experiments were installed in the Bruker TOPSPIN 3.2 pl 6 suite. The free induction decay (FID) acquisition parameters for the standard single pulse test were as follows: (1) (zg30) in ^1^H NMR: spectral width (SW) = 20.64 ppm, relaxation delay (D1) = 1 s, dummy scans (DS) = 2, number of scans (NS) = 16, acquisition time (AQ) = 4.089 s and receiver gain (RG) = 10, giving a total run time of 1 min and 32 s; and (2) (zgpg30) at ^13^C NMR DEPTSP 45: spectral width (SW) = 238.89 ppm, relaxation delay (D1) = 2 s, dummy scans (DS) = 4, number of scans (NS) = 256, acquisition time (AQ) = 1.3631 s and receiver gain (RG) = 2050, giving a total running time of 14 min and 46 s [15]. The ^1^H NMR spectra were processed using exponential multiplication, with a line-broadening factor of 0.3 Hz and a fifth-order polynomial baseline correction (ABSG = 5). Other baseline correction details include: ABSF1 = 10 ppm, ABSF2 = 0 ppm. The ^13^C NMR DEPT 45 experiments were processed using exponential multiplication with a line-broadening factor of 1 Hz (LB = 1 Hz) and a fifth-order polynomial baseline correction (ABSG = 5). Other baseline correction details include: ABSF1 = 170 ppm, ABSF2 = −5 ppm.

Deuterated chloroform 99.8% was obtained from Cambridge Isotope Laboratories, Inc. (Tracer Tec, Madrid, Spain) and used as solvent. Mesitylene 98% was purchased from Sigma-Aldrich (Steinheim, Germany) and used as an internal standard and for normalizing the carbon resonances in the ^13^C spectra. All compounds were used as received, without any purification. All samples were analyzed in quadruplicate.

### 2.3. Statistical Analysis

A multivariate analysis of variance (MANOVA) was used to test if the variables “year”, “variety” or “year × variety” were statistically significant.

A one-way analysis of variance (ANOVA) was used to test differences in NMR signals among different olive oil varieties. The differences between groups were tested using the least significant method (LSD), performed at a 0.05 probability level. After, a linear discriminant analysis (LDA) was performed on data expressing the peaks assigned to olive oils (independent variables) to classify different types of olive oils (grouping variable) according to variety, using only significantly different variables. The statistical significance of each discriminate function was evaluated based on Wilks’ lambda factor. The predictive ability of the LDA model was evaluated by leave-one-out cross-validation.

IBM SPSS Statistics 25 was used to perform all statistical analyses.

## 3. Results and Discussion

### 3.1. NMR Data Interpretation

As previously detailed, ^1^H NMR spectroscopy is the most widely used method for most NMR applications due to the higher sensitivity and lower relaxation times of proton nuclei [16]. However, the ^1^H NMR spectrum contains several overlapping peaks due to the presence of different multiplet patterns that arise from the spin coupling of different protons, which are condensed into a very narrow spectral window (~15 ppm). Additionally, in the ^1^H NMR spectrum, it is not possible to attribute the positional distribution of the fatty acids in the glycerol backbone, constituting a limitation for its widespread use.

As expected, the ^1^H NMR spectra of olive oils show nine resonance signals (Figure S1). The respective chemical shifts and proton assignments are listed in Table 1.

These experiments were carried out under the standard conditions detailed in the experimental section. Analysis of the TGA fraction of one variety of EVOO by ^1^H NMR can be reviewed in our previous publication [15]. These resonance signals are attributed to the fatty acid acyl chain and glyceryl protons of the TGA component. In order to evaluate the composition of olive oils and to differentiate among them, the ^1^H NMR resonance signals were integrated, and mean values for each olive oil variety are presented in Table S1. For the assignment of the ^1^H NMR spectrum, the signal related to the -CH_3_ linolenyl group shows a lower intensity and therefore has not been accounted for in this study. Results from the ANOVA analysis show that only signals S1 and S4 in both years and signal S6 in 2017 are significantly different according to olive oil variety. The largest contribution to the total organic signal obtained for the olive oil samples is given by the 1.22–1.42 ppm signal (S8), which represents the methylene groups of all the fatty acids, followed by the 2.23–2.36 ppm signal (S5) and 1.94–2.14 ppm signal (S6). The lower contribution is given by the 2.70–2.84 ppm signal (S4). As expected, the overlapping of various peaks in the ^1^H NMR spectrum was a problem that limited the potential of this NMR technique.

Thus, the ^13^C NMR technique was attempted in this work in order to provide fast screening for the discrimination of the olive oils under study according to their varietal origin. In fact, at the outset, we looked at the common broadband ^13^C decoupling method and some of the DEPT editing techniques, such as DEPT 90 and DEPT 45. The broadband method was quickly discarded due to the longer accumulation times and lower sensitivity as compared to the DEPT techniques. After comparing the effectiveness of both the DEPT 45 and DEPT 90 methods (which are simple and quick, with good sensitivity), we selected the DEPT 45 method due to the larger number of data points (^13^C signals) compared to the former method (which gives only tertiary ^13^C signals). The ^13^C NMR DEPT 45 spectra of the olive oils under study present 38 characteristic resonances (Figure S2), which are listed with their respective chemical shifts and the corresponding assignments of the carbon signals in Table 2 (detailed assignments were given in a previous publication [15]). The DEPT 45 spectrum of the triglyceride fraction of olive oils shows ^13^C resonances that can be grouped into three defined spectral regions: unsaturated carbons, ranging from 130.20 to 127.92 ppm; glycerol carbons, ranging from 68.91 to 62.10 ppm, and aliphatic carbons, ranging from 34.20 to 14.09 ppm [21,25].

The ^13^C NMR DEPT 45 resonance signals were integrated, and mean values for each olive oil variety are presented in Table S2, where results from the ANOVA analysis are also included. To ensure the highest level of reproducibility and precision in our analysis, it should be noted that peak intensities were normalized through a comparison with the presence of a defined quantity of mesitylene standard (one signal at δ 126.91 ppm), which was added to each NMR tube. The biggest contributions were given by signals S17 and S34, the first being statistically different among the samples in both years. This is followed by signals S10, S12 and S27, which are not significantly different, and S23, S31, S32 and S37, which are significantly different among OO samples, both in 2016 and 2017.

### 3.2. Linear Discriminant Analysis (LDA)

The MANOVA results show that the effects of variety, year and the interaction variety × year are statistically significant. For the ^1^H NMR dataset, Wilks’ lambda is 0.004 (*p* = 0.000) for the effect of variety and 0.412 (*p* = 0.000) for the effect of year, and for the interaction variety × year, it is 0.256 (*p* = 0.000). For the ^13^C NMR DEPT 45 dataset, Wilks’ lambda is 0.15 (*p* = 0.000) for the effect of year, 0.000 (*p* = 0.000) for the effect of variety and 0.004 (*p* = 0.000) for the effect of interaction year × variety. In light of these results, the datasets from 2016 and 2017 were treated separately.

LDA is a supervised pattern recognition method, which produces a set of discriminant functions based on linear combinations of the independent variables, with *a priori* knowledge of each sample group membership. To acquire a more satisfying classification of olive oil samples according to their variety, LDA was performed based on signals exhibiting significant differences (*p* < 0.05) in the ANOVA test. Consequently, eight variables were removed from the original ^13^C NMR DEPT 45 dataset in both years, and seven and six variables were removed from the original ^1^H NMR dataset from 2016 and 2017, respectively.

**Figure 1:**
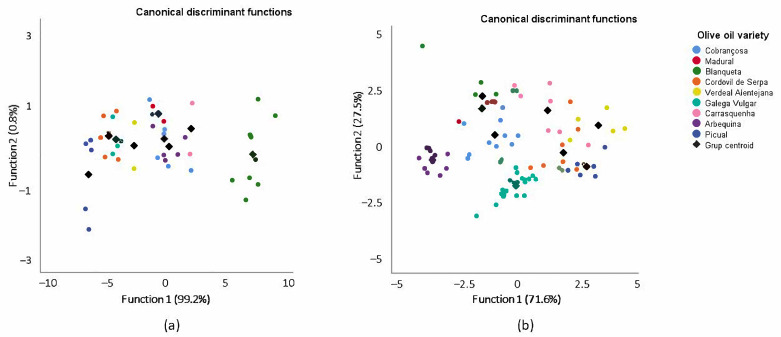
Linear discriminant analysis (represented by canonical discriminant functions) of the olive oils from 2016 (**a**) and 2017 (**b**), categorized into nine groups according to variety, based on significantly different ^1^H NMR signals.

For the ^1^H NMR 2016 dataset, two signals (S1 and S4) were selected, and two discriminant functions were constructed, while for the ^1^H NMR 2017 dataset, three signals (S1, S4 and S6) were selected, and three discriminant functions were constructed, according to the Wilks’ lambda values. Each discriminant function was evaluated for its statistical significance in terms of Wilks’ lambda factor. The range of Wilks’ lambda is from 1 (zero discrimination power) to 0 (perfect discrimination power). For the 2016 dataset, the first two functions explain 100% of the total variance (function 1 explains 99.2% and function 2 explains 0.8%) (Figure 1a), and the Wilks’ lambda values are 0.018 *p* = 0.000) and 0.759 (*p* = 0.200), with a canonical correlation of 0.988 and 0.491, respectively. Thus, only the first function presents good discriminatory power. For the 2017 dataset, function 1 explains 71.6% and function 2 explains 27.5% (Figure 1b) of the total variance. The Wilk’s lambda values are 0.027 (*p* = 0.000) and 0.234 (*p* = 0.000) for the two first functions, with a canonical correlation of 0.939 and 0.862, respectively. For this set of data, both functions, 1 and 2, present good discriminatory power.

In spite of the results from the Mad and Arb samples from 2016, the discriminant functions achieved a satisfactory recognition ability, meaning that 73.8% and 78.3% of olive oil samples from 2016 and 2017 were correctly classified into their correct groups (Table S3) based on the signals of the ^1^H NMR, which presented significant differences among the nine olive oil varieties.

To evaluate the predictive capacity, the generated model was validated by the leave-one-out cross-validation method (Table S3). As a result, 59.5% and 72.3% of olive oil samples from 2016 and 2017 were correctly classified into their groups using this developed model. However, the application of the cross-validation confirmed some misclassification involving samples from Mad 2016 olive oils, where the success of the classification was 0%. In the cases of the Mad (2017), Blaq (2016), Ver (2017), Gal (2017), Carr (2017), Arb and Pic olive oils, the percentage of classification was the same with and without cross-validation in both years.

**Figure 2:**
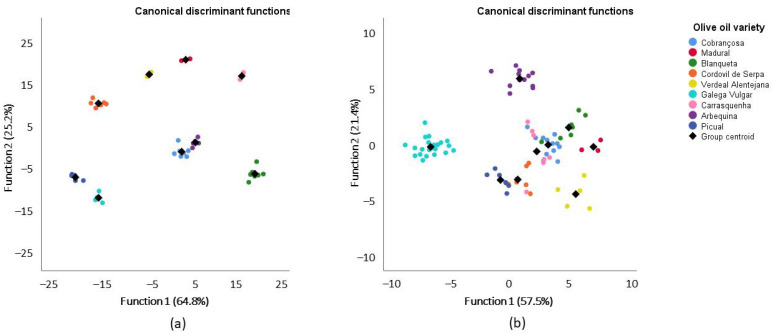
Linear discriminant analysis (represented by canonical discriminant functions) of the olive oils from 2016 (**a**) and 2017 (**b**) categorized into nine groups, according to variety, based on significantly different signals of ^13^C NMR DEPT 45.

For ^13^C NMR DEPT 45, dataset plots of canonical discriminant functions are shown in Figure 2. As stated before, only signals that were significantly different in the ANOVA were used, meaning that linear discriminant analysis was performed without signals on S9, S10, S11, S12, S25, S27, S29 and S34. The two first functions explain 90% (64,8% + 25,2%) of the total variance of olive oil from 2016 (Figure 2a), with a canonical correlation of 0.998 and 0.995, while for olive oils from 2017 (Figure 2b), they explain 78.9% (57,5% + 21,4%), with canonical correlations of 0.980 and 0.948. However, in this case, the first eight functions were considered in the analysis, with significant Wilks’ lambda values for the first four functions (ranging between 0.000 and 0.002 with *p* = 0.000) in 2016 and for the first five functions (ranging from 0.000 to 0.085 with a *p*-value of up to 0.001) in 2017. These results imply that there are significant differences among the olive oils according to their variety.

With the same interpretation as presented before but based on 30 significantly different signals from the ^13^C NMR DEPT 45 (Table S4), the results show that with the 2016 dataset, the recognition ability is 100%, and after cross-validation (CV), it drops to 98.8%, while for the 2017 dataset, it drops from 83.3% to 75.9% after CV. The recognition ability and the predictive capacity of the model is 100% for Gal in both years. The main decreases observed from the recognition ability to the predictive capacity of the model were observed in 2017 for Carr (100% to 28.6%), Ver (100% to 40%) and Blaq (100% to 42.9%) and in 2016 for Arb (100% to 40%) olive oils, which can be explained by the leave-one-out cross-validation approach used. In fact, cross-validation is a technique used to estimate how accurate a predictive model is and gives a more realistic error rate, since in cases with small sample sizes, prediction errors tend to be optimistic.

It is interesting to note that in order to conclude that the model has a good recognition ability, the percentage of correct classification must be bigger than the probability of each case being classified into each group, plus 25%. In our case, the probability of being classified into one of the nine groups is 11.1% + 25% = 36.1%. This value is smaller than the correctly classified percentages shown in Tables S3 and S4 for most of the olive oils. Regardless, it seems that the model based on ^13^C NMR DEPT 45 shows a stronger performance than the model based on ^1^H NMR. It is also interesting to notice that Gal olive oils are the ones with the best performance in the model based on ^13^C NMR DEPT 45. Galega Vulgar olive oils have already proven to be different from other varieties, namely, through their volatile profile [3] and their fatty acid profile [6]. The authors show that by applying a linear discriminant analysis to the fatty acids of olive oils from Alentejo, Galega Vulgar olive oils are very well separated from other olive oil varieties.

## 4. Conclusions

The study developed in this work provided fast screening of varietal origins of olive oils using an NMR spectroscopic-based approach. Indeed, ^13^C NMR DEPT 45 is shown to be a promising tool for the discrimination of olive oils based on their varietal origin, being particularly powerful for the discrimination of olive oils produced from the Galega Vulgar variety. Moreover, the reliability, cost-effectiveness and limited requirements for sample manipulation, without the need for derivatization or pre-treatments, could be regarded as highly advantageous in the application of this analytical methodology for olive oil’s varietal origin labeling.

## Figures and Tables

**Table 1:** Chemical shifts and proton assignments of a ^1^H NMR spectrum of an olive oil sample.

Signal	Chemical Shifts (ppm)	Functional Groups
1	5.26–5.40	-C**H**=C**H**- All unsaturated fatty acids
2	5.20–5.26	>C**H**OCOR Glycerol (triacylglycerols)
3	4.10–4.32	-C**H**_2_OCOR Glycerol (triacylglycerols)
4	2.70–2.84	=CH-C**H**_2_-CH= Linoleyl and linolenyl
5	2.23–2.36	-OCO-C**H**_2_- All acyl chains
6	1.94–2.14	-C**H**_2_-CH=CH- All unsaturated fatty acids
7	1.52–1.70	-OCO-CH_2_-C**H**_2_- All acyl chains
8	1.22–1.42	-(C**H**_2_) _n_ All acyl chains
9	0.83–0.93	-C**H**_3_ All acids except linolenyl group

**Table 2:** Chemical shifts and functional group assignments of a ^13^C DEPT45 spectrum of an olive oil sample.

Signal	Chemical Shifts (ppm)	Functional Groups	Signal	Chemical Shifts (ppm)	Functional Groups
1	130.20	L13 αβ	20	29.57	O14 αβ
2	130.01	O 10 αβ	21	29.52	S6 α
3	129.93	L9 α	22	29.41	S15 α
4	129.83	L9β	23	29.36	L15 αβ
5	129.71	O9 α	24	29.31	O15,13 αβ
6	129.69	O9 β	25	29.23	S5 α
7	128.10	L10 αβ	26	29.21	O, L5 β
8	127.92	L12 αβ	27	29.14	O, L5 α
9	68.91	Gl β	28	29.12	S4 α-O, L6 α, β-O, L4 α
10	62.10	Gl α	29	29.08	O, L4 β
11	34.20	O, L 2 β	30	29.02	unknown
12	34.04	S2 α	31	27.25	O11 αβ
13	31.95	S16 α/O16 αβ	32	27.20	L8 αβ, O8 αβ
14	31.82	unknown	33	25.65	L11 αβ
15	31.56	L16 αβ	34	24.87	O, L3 αβ, S3 α
16	29.80	O12 αβ	35	22.72	S17 α, O17 αβ
17	29.74	unknown	36	22.61	L17 αβ
18	29.70	unknown	37	14.13	S18 α, O18 αβ
19	29.66	unknown	38	14.09	L18 αβ

## Data Availability

Not applicable.

## Supplementary Materials

The following are available online at https://www.mdpi.com/2304-8158/10/2/399/s1, Figure S1: ^1^H NMR spectrum (400 MHz in CDCl_3_) of the monovarietal Portuguese olive oil Cobrançosa cv. The referencing of ^1^H chemical shifts have been done by using the H- signal of the residual CHCl_3_ in the CDCl_3_ solvent (S assignment)). Figure S2: ^13^C NMR DEPT45 spectrum (100.13 MHz in CDCl_3_) of the monovarietal Portuguese Olive Oil Cobrançosa cv (IS—internal standard). The referencing of ^13^C chemical shifts have been done by using carbon-13 shift of the CDCl_3_ solvent. Table S1: Average value for each ^1^H NMR signal, for each olive oil variety, Wilks’ Lambda and *p*-value from ANOVA analysis with the LSD method at *p* < 0.05; Table S2: Average value for each ^13^C NMR DEPT 45 signal, for each olive oil variety, Wilks’ Lambda and *p*-value from ANOVA analysis with the LSD method at *p* < 0.05; Table S3: Classification results of olive oil samples and percentage of observations correctly classified by LDA, based on significantly different ^1^H NMR signal of olive oils from nine varieties; Table S4: Classification results of olive oil samples and percentage of observations correctly classified by LDA, based on significantly different ^13^C NMR DEPT 45 signals of olive oils from nine varieties.
