# Supplementary figures and images for: Seeking a Fast Screening Method of the Varietal Origin of Olive Oil: The Usefulness of an NMR-Based Approach
Source: Foods. 2021 Feb 11;10(2):399. doi: 10.3390/foods10020399 (PMC7918584; doi:10.3390/foods10020399)

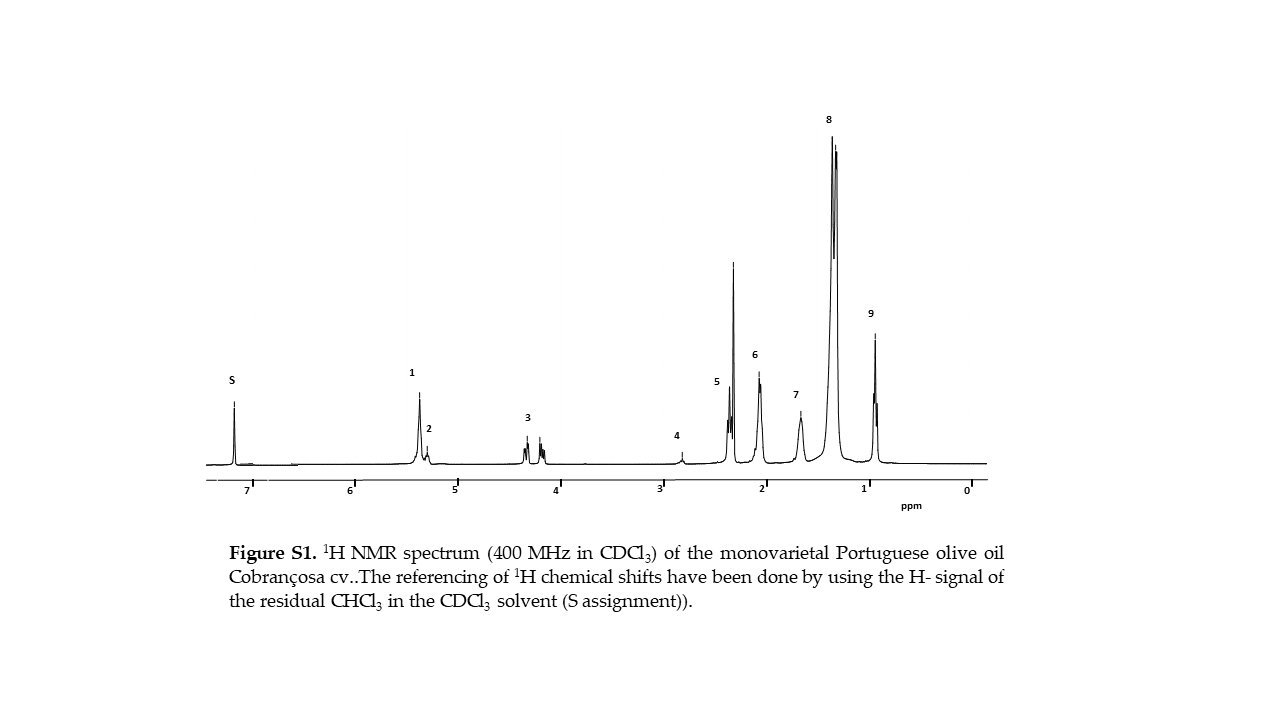

Supplement: Supplementary file 1 [file foods-10-00399-s001.zip › foods-1094696- supplementary/Figure S1_1H NMR_new.tif]

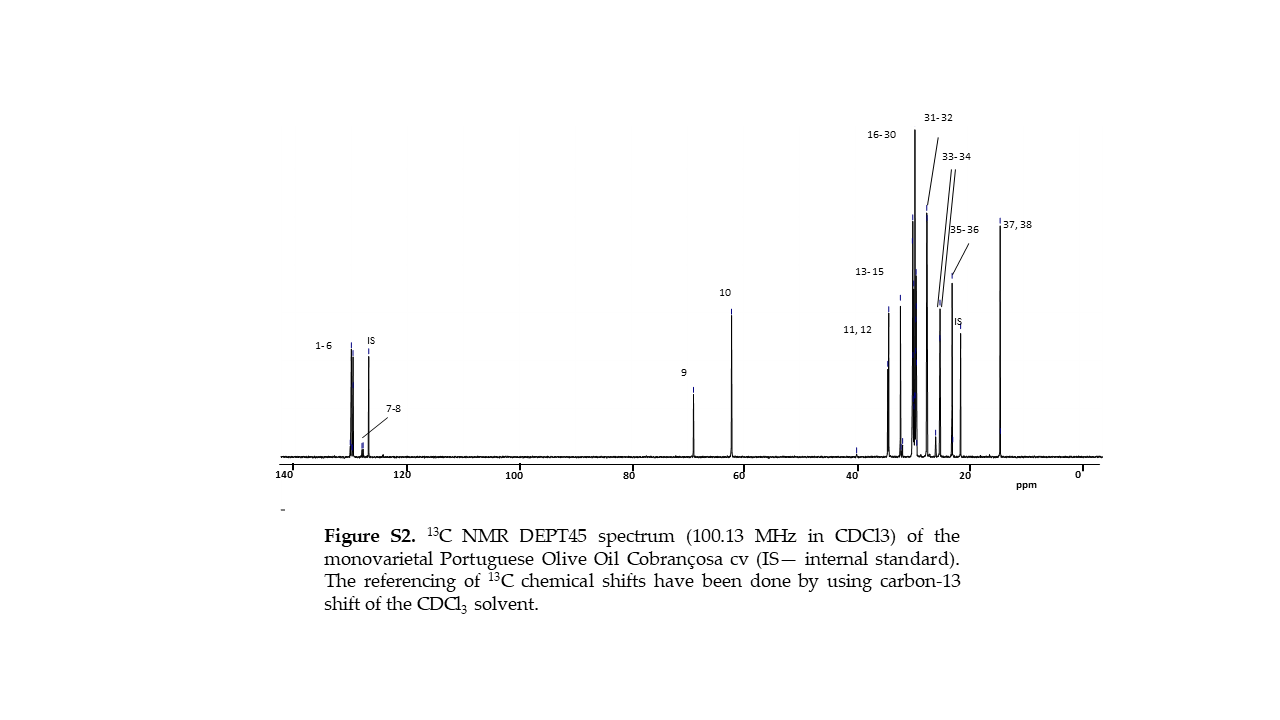

Supplement: Supplementary file 1 [file foods-10-00399-s001.zip › foods-1094696- supplementary/Figure S2_13C NMR DEPT45_new.tif]
