# Supplementary material for: Seeking a Fast Screening Method of the Varietal Origin of Olive Oil: The Usefulness of an NMR-Based Approach
Source: Foods. 2021 Feb 11;10(2):399. doi: 10.3390/foods10020399 (PMC7918584; doi:10.3390/foods10020399)
Supplement: Supplementary file 1 [file foods-10-00399-s001.zip › foods-1094696- supplementary/foods-1094696-table sup.zip!/Table S1.docx]

**Table S1 –** Average value for each ^1^H NMR signal, for each olive oil variety, Wilks’ Lambda and *p*-value from ANOVA analysis with LSD method at p<0.05

| Signal | Olive Oil Variety - 2016 | | | | | | | | |  |  |
| --- | --- | --- | --- | --- | --- | --- | --- | --- | --- | --- | --- |
|  | **Cob**  **(n=7)** | **Mad (n=2)** | **Blaq**  **(n=9)** | **Cor**  **(n=6)** | **Ver (n=2)** | **Gal (n=4)** | **Carr (n=2)** | **Arb**  **(n=5)** | **Pic**  **(n=5)** | **Wilks’ Lambda** | ***p*-value** |
| 1 | 3.3050 | 3.4013 | 3.3706 | 3.2379 | 3.2338 | 3.2325 | 3.3875 | 3.2800 | 3.0460 | 0.526 | 0.003^a^ |
| 2 | 1.0050 | 0.9825 | 0.9942 | 0.9921 | 0.9713 | 1.0256 | 1.0225 | 0.9885 | 0.9590 | 0.784 | 0.367^b^ |
| 3 | 1.0100 | 1.0000 | 0.9931 | 0.9946 | 0.9713 | 1.0250 | 1.0150 | 0.9855 | 0.9450 | 0.706 | 0.131^b^ |
| 4 | 0.2925 | 0.2825 | 0.5092 | 0.1575 | 0.2188 | 0.1750 | 0.3612 | 0.3040 | 0.1020 | 0.025 | 0.000^a^ |
| 5 | 6.4196 | 6.4338 | 6.4142 | 6.4279 | 6.3813 | 6.4937 | 6.6625 | 6.4055 | 6.3215 | 0.790 | 0.390^b^ |
| 6 | 5.2418 | 5.4675 | 5.0817 | 5.4546 | 5.3650 | 5.3075 | 5.3888 | 5.1695 | 5.1810 | 0.674 | 0.078^b^ |
| 7 | 3.4604 | 3.3762 | 3.4669 | 3.4450 | 3.4300 | 3.5031 | 3.6225 | 3.3260 | 3.3065 | 0.742 | 0.218^b^ |
| 8 | 32.4236 | 31.9113 | 31.2775 | 32.0792 | 31.2425 | 33.0825 | 32.5750 | 31.8240 | 31.1175 | 0.747 | 0.234^b^ |
| 9 | 4.8004 | 4.7600 | 4.7439 | 4.7417 | 4.6350 | 4.8794 | 4.8350 | 4.6775 | 4.6535 | 0.814 | 0.497^b^ |
|  | **Olive Oil Variety – 2017** | | | | | | | | |  |  |
|  | **Cob (n=12)** | **Mad (n=3)** | **Blaq**  **(n=7)** | **Cor**  **(n=9)** | **Ver (n=5)** | **Gal (n=22)** | **Carr (n=7)** | **Arb (n=12)** | **Pic**  **(n=6)** | **Wilks’ Lambda** | ***p*-value** |
| 1 | 3.1858 | 3.3850 | 3.2446 | 3.1053 | 3.1690 | 2.9945 | 3.1889 | 3.1590 | 3.0571 | 0.602 | 0.000^a^ |
| 2 | 0.9827 | 0.9775 | 0.9693 | 0.9636 | 0.9675 | 0.9734 | 0.9643 | 0.9681 | 0.9717 | 0.970 | 0.968^b^ |
| 3 | 0.9696 | 0.9792 | 0.9554 | 0.9564 | 0.9580 | 0.9599 | 0.9579 | 0.9548 | 0.9550 | 0.969 | 0.965^b^ |
| 4 | 0.3452 | 0.4583 | 0.4671 | 0.2072 | 0.2450 | 0.1630 | 0.3457 | 0.3517 | 0.1375 | 0.232 | 0.000^a^ |
| 5 | 6.3413 | 6.3225 | 6.2318 | 6.2711 | 6.2815 | 6.2878 | 6.2714 | 6.2642 | 6.3054 | 0.943 | 0.809^b^ |
| 6 | 4.9823 | 5.1467 | 4.9014 | 5.1358 | 5.2490 | 4.9326 | 5.0700 | 4.8292 | 5.1829 | 0.637 | 0.000^a^ |
| 7 | 3.3629 | 3.3300 | 3.3207 | 3.3531 | 3.4035 | 3.2997 | 3.3911 | 3.1944 | 3.4138 | 0.768 | 0.009^b^ |
| 8 | 30.9296 | 30.4050 | 29.7686 | 30.6808 | 30.7460 | 31.1684 | 30.4361 | 30.3846 | 31.1992 | 0.855 | 0.150^b^ |
| 9 | 4.5913 | 4.5867 | 4.4768 | 4.5522 | 4.5470 | 4.5752 | 4.5525 | 4.5354 | 4.5483 | 0.963 | 0.938^b^ |

^a^ There is significant differences for this signal among the nine olive oil varieties; ^b^ There is no significant differences for this signal among the nine olive oil varieties. Cob - Cobrançosa; Mad - Madural; Blaq - Blanqueta; Cor - Cordovil de Serpa; Ver - Verdeal Alentejana; Gal - Galega Vulgar; Carr - Carrasquenha; Arb - Arbequina; Pic - Picual
