# Supplementary material for: Seeking a Fast Screening Method of the Varietal Origin of Olive Oil: The Usefulness of an NMR-Based Approach
Source: Foods. 2021 Feb 11;10(2):399. doi: 10.3390/foods10020399 (PMC7918584; doi:10.3390/foods10020399)
Supplement: Supplementary file 1 [file foods-10-00399-s001.zip › foods-1094696- supplementary/foods-1094696-table sup.zip!/Table S2.docx]

**Table S2 –** Average value for each ^13^C NMR DEPT 45 signal, for each olive oil variety, Wilks’ Lambda and *p*-value from ANOVA analysis with LSD method at p<0.05

| Signal | Olive Oil Variety - 2016 | | | | | | | | |  |  |
| --- | --- | --- | --- | --- | --- | --- | --- | --- | --- | --- | --- |
|  | **Cob**  **(n=7)** | **Mad (n=2)** | **Blaq**  **(n=9)** | **Cor**  **(n=6)** | **Ver (n=2)** | **Gal (n=4)** | **Carr (n=2)** | **Arb**  **(n=5)** | **Pic**  **(n=5)** | **Wilks’ Lambda** | ***p*-value** |
| 1 | 0.2136 | 0.2163 | 0.3267 | 0.1308 | 0.1750 | 0.1263 | 0.2613 | 0.2225 | 0.0920 | 0.040 | 0.000^a^ |
| 2 | 1.5618 | 1.6538 | 1.4858 | 1.6154 | 1.6375 | 1.5569 | 1.6938 | 1.5635 | 1.5875 | 0.559 | 0.008^a^ |
| 3 | 0.0804 | 0.0600 | 0.0875 | 0.0796 | 0.0650 | 0.1131 | 0.0938 | 0.0905 | 0.0765 | 0.341 | 0.000^a^ |
| 4 | 0.0729 | 0.0625 | 0.0767 | 0.0700 | 0.0588 | 0.0994 | 0.0850 | 0.0910 | 0.0705 | 0.288 | 0.000^a^ |
| 5 | 0.7957 | 0.8800 | 0.6633 | 0.8987 | 0.8600 | 0.8206 | 0.8425 | 0.8030 | 0.8655 | 0.226 | 0.000^a^ |
| 6 | 0.6032 | 0.6075 | 0.5192 | 0.6379 | 0.6513 | 0.6713 | 0.6188 | 0.5610 | 0.6455 | 0.316 | 0.000^a^ |
| 7 | 0.1429 | 0.1350 | 0.2692 | 0.0538 | 0.1063 | 0.0688 | 0.1838 | 0.1615 | 0.0335 | 0.025 | 0.000^a^ |
| 8 | 0.1307 | 0.1263 | 0.2444 | 0.0504 | 0.1000 | 0.0694 | 0.1700 | 0.1465 | 0.0370 | 0.024 | 0.000^a^ |
| 9 | 0.7789 | 0.7688 | 0.7658 | 0.7650 | 0.7575 | 0.7906 | 0.8813 | 0.7735 | 0.7655 | 0.625 | 0.032^b^ |
| 10 | 2.2818 | 2.2562 | 2.2850 | 2.2529 | 2.2938 | 2.3319 | 2.6213 | 2.3235 | 2.2675 | 0.659 | 0.060^b^ |
| 11 | 1.1025 | 1.1013 | 1.1083 | 1.0929 | 1.1175 | 1.1250 | 1.2487 | 1.1105 | 1.0990 | 0.723 | 0.168^b^ |
| 12 | 2.1968 | 2.1700 | 2.1981 | 2.1738 | 2.2013 | 2.2344 | 2.4938 | 2.2335 | 2.1670 | 0.675 | 0.079^b^ |
| 13 | 1.6679 | 1.6888 | 1.5242 | 1.7275 | 1.7000 | 1.7181 | 1.7188 | 1.6195 | 1.7215 | 0.461 | 0.001^a^ |
| 14 | 0.0975 | 0.0663 | 0.1075 | 0.0896 | 0.0700 | 0.1556 | 0.1375 | 0.1210 | 0.0870 | 0.162 | 0.000^a^ |
| 15 | 0.1571 | 0.1463 | 0.2669 | 0.0729 | 0.1137 | 0.0900 | 0.1913 | 0.1660 | 0.0555 | 0.020 | 0.000^a^ |
| 16 | 1.8375 | 1.9663 | 1.5883 | 2.0196 | 2.0088 | 1.9294 | 1.9775 | 1.8015 | 2.0110 | 0.249 | 0.000^a^ |
| 17 | 3.3829 | 3.1250 | 3.2467 | 3.2263 | 3.2512 | 3.5125 | 3.7213 | 3.3185 | 3.3370 | 0.589 | 0.015^a^ |
| 18 | 1.0043 | 0.7925 | 1.1294 | 0.8546 | 0.8113 | 1.0350 | 1.0700 | 0.9800 | 0.8610 | 0.098 | 0.000^a^ |
| 19 | 0.8800 | 0.7650 | 1.1242 | 0.6638 | 0.7313 | 0.7981 | 1.0363 | 0.9030 | 0.6390 | 0.039 | 0.000^a^ |
| 20 | 1.5561 | 1.6375 | 1.3508 | 1.7042 | 1.6600 | 1.6388 | 1.6713 | 1.5475 | 1.7025 | 0.254 | 0.000^a^ |
| 21 | 0.5104 | 0.4025 | 0.5614 | 0.4262 | 0.4350 | 0.5169 | 0.5750 | 0.5080 | 0.4410 | 0.126 | 0.000^a^ |
| 22 | 0.5204 | 0.4450 | .5700 | 0.4558 | 0.4425 | 0.5538 | 0.5663 | 0.5205 | 0.4805 | 0.229 | 0.000^a^ |
| 23 | 3.0043 | 3.2138 | 2.7156 | 3.2033 | 3.2063 | 2.9894 | 3.1887 | 2.9525 | 3.1390 | 0.360 | 0.000^a^ |
| 24 | 0.7425 | 0.6013 | 0.8269 | 0.6463 | 0.6375 | 0.8431 | 0.8763 | 0.7750 | 0.6695 | 0.147 | 0.000^a^ |
| 25 | 0.8107 | 0.7813 | 0.7897 | 0.8033 | 0.8463 | 0.8194 | 0.9225 | 0.8235 | 0.8051 | 0.739 | 0.212^b^ |
| 26 | 1.1468 | 1.2613 | 1.0767 | 1.2088 | 1.1925 | 1.1312 | 1.3413 | 1.1430 | 1.1680 | 0.424 | 0.000^a^ |
| 27 | 2.3654 | 2.3200 | 2.3653 | 2.3438 | 2.3850 | 2.4031 | 2.6925 | 2.4005 | 2.3110 | 0.662 | 0.064^b^ |
| 28 | 1.2843 | 1.4200 | 1.2167 | 1.3254 | 1.3788 | 1.2744 | 1.4050 | 1.2915 | 1.3220 | 0.578 | 0.012^a^ |
| 29 | 0.9604 | 0.9575 | 0.9572 | 0.9700 | 0.9538 | 0.9888 | 1.0975 | 0.9800 | 0.9625 | 0.717 | 0.154^b^ |
| 30 | 0.1914 | 0.1575 | 0.2108 | 0.1754 | 0.1600 | 0.2494 | 0.2325 | 0.2180 | 0.1540 | 0.276 | 0.000^a^ |
| 31 | 2.5414 | 2.6363 | 1.9117 | 2.6221 | 2.6200 | 2.6044 | 2.8525 | 2.6455 | 2.5875 | 0.234 | 0.000^a^ |
| 32 | 2.4782 | 2.6313 | 2.0058 | 2.6000 | 2.6925 | 2.5206 | 2.7813 | 2.5100 | 2.5810 | 0.204 | 0.000^a^ |
| 33 | 0.3164 | 0.3000 | 0.5364 | 0.1875 | 0.2725 | 0.1963 | 0.4225 | 0.3295 | 0.1425 | 0.026 | 0.000^a^ |
| 34 | 3.2411 | 3.1863 | 3.2447 | 3.1829 | 3.2213 | 3.3031 | 3.6663 | 3.2600 | 3.1615 | 0.673 | 0.078^b^ |
| 35 | 1.8975 | 1.8738 | 1.7503 | 1.9058 | 1.8650 | 2.0025 | 1.9138 | 1.8465 | 1.9100 | 0.548 | 0.006^a^ |
| 36 | 0.2046 | 0.1775 | 0.3208 | 0.1125 | 0.1500 | 0.1244 | 0.2413 | 0.2155 | 0.0735 | 0.034 | 0.000^a^ |
| 37 | 2.1750 | 2.1838 | 2.0172 | 2.2488 | 2.1738 | 2.3069 | 2.1963 | 2.1505 | 2.2290 | 0.466 | 0.001^a^ |
| 38 | 0.3007 | 0.2800 | 0.4500 | 0.1825 | 0.2450 | 0.2081 | 0.3562 | 0.3220 | 0.2115 | 0.139 | 0.000^a^ |
|  |  |  |  |  |  |  |  |  |  |  |  |
| Signal | **Olive Oil Variety - 2017** | | | | | | | | |  |  |
|  | **Cob**  **(n=12)** | **Mad (n=3)** | **Blaq**  **(n=7)** | **Cor**  **(n=9)** | **Ver (n=5)** | **Gal (n=22)** | **Carr (n=7)** | **Arb**  **(n=12)** | **Pic**  **(n=6)** | **Wilks’ Lambda** | ***p*-value** |
| 1 | 0.2250 | 0.2858 | 0.2982 | 0.1564 | 0.1770 | 0.1256 | 0.2339 | 0.2440 | 0.1150 | 0.272 | 0.000^a^ |
| 2 | 1.5248 | 1.5617 | 1.4621 | 1.5417 | 1.5870 | 1.4701 | 1.4875 | 1.4735 | 1.5458 | 0.716 | 0.001^a^ |
| 3 | 0.0779 | 0.0750 | 0.0786 | 0.0747 | 0.0695 | 0.0891 | 0.0836 | 0.0892 | 0.0829 | 0.665 | 0.000^a^ |
| 4 | 0.0658 | 0.0633 | 0.0696 | 0.0692 | 0.0590 | 0.0820 | 0.0739 | 0.0821 | 0.0692 | 0.496 | 0.000^a^ |
| 5 | 0.7615 | 0.7758 | 0.6989 | 0.8192 | 0.8665 | 0.7923 | 0.7614 | 0.7437 | 0.8342 | 0.520 | 0.000^a^ |
| 6 | 0.5685 | 0.5658 | 0.5132 | 0.6053 | 0.5915 | 0.6016 | 0.5425 | 0.5127 | 0.6463 | 0.479 | 0.000^a^ |
| 7 | 0.1710 | 0.2325 | 0.2475 | 0.0878 | 0.1135 | 0.0659 | 0.1754 | 0.1863 | 0.0479 | 0.229 | 0.000^a^ |
| 8 | 0.1577 | 0.2158 | 0.2254 | 0.0836 | 0.1080 | 0.0625 | 0.1614 | 0.1744 | 0.0513 | 0.217 | 0.000^a^ |
| 9 | 0.7710 | 0.7617 | 0.7443 | 0.7475 | 0.7580 | 0.7537 | 0.7486 | 0.7488 | 0.7604 | 0.947 | 0.838^b^ |
| 10 | 2.2833 | 2.2217 | 2.1857 | 2.1989 | 2.2160 | 2.2009 | 2.1843 | 2.2002 | 2.1408 | 0.896 | 0.394^b^ |
| 11 | 1.0977 | 1.0733 | 1.0536 | 1.0669 | 1.0805 | 1.0608 | 1.0521 | 1.0623 | 1.0783 | 0.921 | 0.614^b^ |
| 12 | 2.1804 | 2.1392 | 2.0879 | 2.1125 | 2.1275 | 2.1062 | 2.0979 | 2.1269 | 2.1287 | 0.927 | 0.662^b^ |
| 13 | 1.5988 | 1.5333 | 1.4646 | 1.6483 | 1.6500 | 1.6415 | 1.5421 | 1.5306 | 1.6950 | 0.466 | 0.000^a^ |
| 14 | 0.0910 | 0.0808 | 0.0893 | 0.0961 | 0.0740 | 0.1261 | 0.1061 | 0.1148 | 0.1029 | 0.274 | 0.000^a^ |
| 15 | 0.1754 | 0.2242 | 0.2393 | 0.1006 | 0.1225 | 0.0786 | 0.1800 | 0.1892 | 0.0688 | 0.226 | 0.000^a^ |
| 16 | 1.7281 | 1.7325 | 1.5732 | 1.8825 | 1.8830 | 1.7978 | 1.6921 | 1.6535 | 1.9304 | 0.352 | 0.000^a^ |
| 17 | 3.3625 | 3.0767 | 3.0732 | 3.1967 | 3.1950 | 3.3820 | 3.1671 | 3.2069 | 3.3033 | 0.638 | 0.000^a^ |
| 18 | 1.0513 | 0.8842 | 1.0089 | 0.8997 | 0.8580 | 1.0223 | 0.9696 | 1.0127 | 0.9267 | 0.567 | 0.000^a^ |
| 19 | 0.9525 | 0.9550 | 1.0211 | 0.7389 | 0.7485 | 0.7750 | 0.8975 | 0.9427 | 0.6854 | 0.362 | 0.000^a^ |
| 20 | 1.4788 | 1.4650 | 1.3332 | 1.5828 | 1.6040 | 1.5417 | 1.4404 | 1.4275 | 1.6412 | 0.384 | 0.000^a^ |
| 21 | 0.5256 | 0.4383 | 0.5104 | 0.4558 | 0.4310 | 0.5136 | 0.4911 | 0.5077 | 0.4654 | 0.517 | 0.000^a^ |
| 22 | 0.5340 | 0.4808 | 0.5221 | 0.4753 | 0.4580 | 0.5323 | 0.5118 | 0.5242 | 0.4846 | 0.556 | 0.000^a^ |
| 23 | 2.8719 | 2.9108 | 2.6632 | 2.9969 | 3.0740 | 2.8294 | 2.7882 | 2.7435 | 3.0542 | 0.492 | 0.000^a^ |
| 24 | 0.7617 | 0.6692 | 0.7229 | 0.6742 | 0.6445 | 0.7869 | 0.7346 | 0.7744 | 0.6992 | 0.521 | 0.000^a^ |
| 25 | 0.7994 | 0.7942 | 0.7661 | 0.7850 | 0.8195 | 0.7693 | 0.7682 | 0.7788 | 0.7813 | 0.901 | 0.429^b^ |
| 26 | 1.1094 | 1.1625 | 1.0439 | 1.1325 | 1.1465 | 1.0514 | 1.0846 | 1.0538 | 1.1329 | 0.630 | 0.000^a^ |
| 27 | 2.3688 | 2.3292 | 2.2729 | 2.2792 | 2.2990 | 2.2882 | 2.2704 | 2.2896 | 2.3246 | 0.922 | 0.615^b^ |
| 28 | 1.2362 | 1.2875 | 1.1800 | 1.2756 | 1.3275 | 1.1857 | 1.2075 | 1.1813 | 1.2379 | 0.593 | 0.000^a^ |
| 29 | 0.9629 | 0.9400 | 0.9093 | 0.9267 | 0.9370 | 0.9252 | 0.9232 | 0.9213 | 0.9488 | 0.901 | 0.435^b^ |
| 30 | 0.1800 | 0.1533 | 0.1654 | 0.1611 | 0.1490 | 0.1983 | 0.1832 | 0.1925 | 0.1771 | 0.582 | 0.000^a^ |
| 31 | 1.9885 | 1.9767 | 1.7839 | 2.4356 | 2.2795 | 2.4074 | 2.0057 | 1.9194 | 2.5225 | 0.190 | 0.000^a^ |
| 32 | 2.2971 | 2.1033 | 1.9482 | 2.4408 | 2.5325 | 2.3350 | 2.1868 | 2.1675 | 2.4558 | 0.529 | 0.000^a^ |
| 33 | 0.3702 | 0.4583 | 0.4846 | 0.2325 | 0.2790 | 0.1832 | 0.3729 | 0.3567 | 0.1758 | 0.245 | 0.000^a^ |
| 34 | 3.1967 | 3.1367 | 3.0611 | 3.0869 | 3.1210 | 3.0818 | 3.0775 | 3.1027 | 3.1188 | 0.924 | 0.640^b^ |
| 35 | 1.7963 | 1.7158 | 1.6607 | 1.8536 | 1.8405 | 1.8802 | 1.7532 | 1.7544 | 1.9021 | 0.458 | 0.000^a^ |
| 36 | 0.2215 | 0.2675 | 0.2846 | 0.1361 | 0.1610 | 0.1185 | 0.2218 | 0.2363 | 0.1071 | 0.212 | 0.000^a^ |
| 37 | 2.0579 | 1.9692 | 1.9082 | 2.1317 | 2.1210 | 2.1636 | 2.0189 | 2.0150 | 2.2025 | 0.438 | 0.000^a^ |
| 38 | 0.3144 | 0.3667 | 0.3886 | 0.2128 | 0.2505 | 0.1916 | 0.3136 | 0.3404 | 0.1763 | 0.225 | 0.000^a^ |

^a^ There is significant differences for this signal among the nine olive oil varieties; ^b^ There is no significant differences for this signal among the nine olive oil varieties. Cob - Cobrançosa; Mad - Madural; Blaq - Blanqueta; Cor - Cordovil de Serpa; Ver - Verdeal Alentejana; Gal - Galega Vulgar; Carr - Carrasquenha; Arb - Arbequina; Pic - Picual
