# Supplementary material for: Seeking a Fast Screening Method of the Varietal Origin of Olive Oil: The Usefulness of an NMR-Based Approach
Source: Foods. 2021 Feb 11;10(2):399. doi: 10.3390/foods10020399 (PMC7918584; doi:10.3390/foods10020399)
Supplement: Supplementary file 1 [file foods-10-00399-s001.zip › foods-1094696- supplementary/foods-1094696-table sup.zip!/Table S3.docx]

**Table S3 -** Classification results of olive oil samples and percentage of observations correctly classified by LDA, based on significantly different ^1^H NMR signal of olive oils from 9 varieties

| **Variety** | **CV** | **Nº of samples** | **Predicted Group Membership** | | | | | | | | | **Correctly Classified (%)** |
| --- | --- | --- | --- | --- | --- | --- | --- | --- | --- | --- | --- | --- |
|  |  |  | **Cob** | **Mad** | **Blaq** | **Cor** | **Ver** | **Gal** | **Carr** | **Arb** | **Pic** |  |
| **Cob**  2016 | no | 7 | 5 | 1 | 0 | 0 | 0 | 0 | 1 | 0 | 0 | 71.4 |
|  | yes |  | 3 | 2 | 0 | 0 | 0 | 0 | 1 | 1 | 0 | 42.9 |
| **Cob**  2017 | no  yes | 12 | 9  6 | 1  1 | 1  2 | 0  0 | 0  0 | 1  1 | 0  0 | 0  2 | 0  0 | 75.0  50.0 |
| **Mad**  2016 | no | 2 | 2 | 0 | 0 | 0 | 0 | 0 | 0 | 0 | 0 | 0 |
|  | yes |  | 2 | 0 | 0 | 0 | 0 | 0 | 0 | 0 | 0 | 0 |
| **Mad** | no | 3 | 1 | 2 | 0 | 0 | 0 | 0 | 0 | 0 | 0 | 66.7 |
| 2017 | yes |  | 1 | 2 | 0 | 0 | 0 | 0 | 0 | 0 | 0 | 66.7 |
| **Blaq**  2016 | no | 9 | 0 | 0 | 9 | 0 | 0 | 0 | 0 | 0 | 0 | 100 |
|  | yes |  | 0 | 0 | 9 | 0 | 0 | 0 | 0 | 0 | 0 | 100 |
| **Blaq** | no | 7 | 0 | 0 | 6 | 0 | 0 | 1 | 0 | 0 | 0 | 85.7 |
| 2017 | yes |  | 0 | 1 | 5 | 0 | 0 | 1 | 0 | 0 | 0 | 71.4 |
| **Cor**  2016 | no | 6 | 0 | 0 | 0 | 5 | 0 | 1 | 0 | 0 | 0 | 83.3 |
|  | yes |  | 0 | 0 | 0 | 4 | 0 | 2 | 0 | 0 | 0 | 66.7 |
| **Cor** | no | 9 | 0 | 0 | 0 | 4 | 0 | 2 | 1 | 0 | 2 | 44.4 |
| 2017 | yes |  | 0 | 0 | 0 | 3 | 1 | 2 | 1 | 0 | 2 | 33.3 |
| **Ver**  2016 | no | 2 | 0 | 0 | 0 | 0 | 2 | 0 | 0 | 0 | 0 | 100 |
|  | yes |  | 0 | 0 | 0 | 0 | 1 | 1 | 0 | 0 | 0 | 50.0 |
| **Ver** | no | 5 | 0 | 0 | 0 | 1 | 4 | 0 | 0 | 0 | 0 | 80.0 |
| 2017 | yes |  | 0 | 0 | 0 | 1 | 4 | 0 | 0 | 0 | 0 | 80.0 |
| **Gal**  2016 | no | 4 | 0 | 0 | 0 | 2 | 0 | 2 | 0 | 0 | 0 | 50.0 |
|  | yes |  | 0 | 0 | 0 | 3 | 0 | 1 | 0 | 0 | 0 | 25.0 |
| **Gal** | no | 22 | 0 | 0 | 0 | 1 | 0 | 21 | 0 | 0 | 0 | 95.5 |
| 2017 | yes |  | 0 | 0 | 0 | 1 | 0 | 21 | 0 | 0 | 0 | 95.5 |
| **Carr**  2016 | no | 2 | 0 | 0 | 0 | 0 | 0 | 0 | 2 | 0 | 0 | 100 |
|  | yes |  | 0 | 0 | 0 | 0 | 0 | 0 | 1 | 1 | 0 | 50.0 |
| **Carr** | no | 7 | 0 | 0 | 1 | 2 | 0 | 0 | 4 | 0 | 0 | 57.1 |
| 2017 | yes |  | 0 | 0 | 1 | 2 | 0 | 0 | 4 | 0 | 0 | 57.1 |
| **Arb**  2016 | no | 5 | 3 | 0 | 0 | 0 | 0 | 0 | 1 | 1 | 0 | 20.0 |
|  | yes |  | 3 | 0 | 0 | 0 | 0 | 0 | 1 | 1 | 0 | 20.0 |
| **Arb** | no | 12 | 0 | 0 | 0 | 0 | 0 | 0 | 0 | 12 | 0 | 100 |
| 2017 | yes |  | 0 | 0 | 0 | 0 | 0 | 0 | 0 | 12 | 0 | 100 |
| **Pic**  2016 | no | 5 | 0 | 0 | 0 | 0 | 0 | 0 | 0 | 0 | 5 | 100 |
|  | yes |  | 0 | 0 | 0 | 0 | 0 | 0 | 0 | 0 | 5 | 100 |
| **Pic** | no | 6 | 0 | 0 | 0 | 2 | 1 | 0 | 0 | 0 | 3 | 50.0 |
| 2017 | yes |  | 0 | 0 | 0 | 2 | 1 | 0 | 0 | 0 | 3 | 50.0 |
| **Total**  2016 | no | 42 | 10 | 1 | 9 | 7 | 2 | 3 | 4 | 1 | 5 | 73.8 |
|  | yes |  | 8 | 2 | 9 | 7 | 1 | 4 | 3 | 3 | 5 | 59.5 |
| **Total** | no | 83 | 10 | 3 | 8 | 10 | 5 | 25 | 5 | 12 | 5 | 78.3 |
| 2017 | yes |  | 7 | 4 | 8 | 9 | 6 | 25 | 5 | 14 | 5 | 72.3 |

Cob - Cobrançosa; Mad - Madural; Blaq - Blanqueta; Cor - Cordovil de Serpa; Ver -Verdeal Alentejana; Gal - Galega Vulgar; Carr - Carrasquenha; Arb - Arbequina; Pic - Picual. CV - Cross validation
