# Supplementary material for: Seeking a Fast Screening Method of the Varietal Origin of Olive Oil: The Usefulness of an NMR-Based Approach
Source: Foods. 2021 Feb 11;10(2):399. doi: 10.3390/foods10020399 (PMC7918584; doi:10.3390/foods10020399)
Supplement: Supplementary file 1 [file foods-10-00399-s001.zip › foods-1094696- supplementary/foods-1094696-table sup.zip!/Table S4.docx]

**Table S4 -** Classification results of olive oil samples and percentage of observations correctly classified by LDA, based on significantly different ^13^C NMR DEPT 45 signal of olive oils from 9 varieties

| **Variety** | **CV** | **Nº of samples** | **Predicted Group Membership** | | | | | | | | | **Correctly Classified (%)** |
| --- | --- | --- | --- | --- | --- | --- | --- | --- | --- | --- | --- | --- |
|  |  |  | **Cob** | **Mad** | **Blaq** | **Cor** | **Ver** | **Gal** | **Carr** | **Arb** | **Pic** |  |
| **Cob**  2016 | no | 7 | 7 | 0 | 0 | 0 | 0 | 0 | 0 | 0 | 0 | 100 |
|  | yes |  | 5 | 0 | 0 | 0 | 0 | 0 | 0 | 2 | 0 | 71.4 |
| **Cob**  2017 | no  yes | 12 | 12  9 | 0  0 | 0  1 | 0  0 | 0  0 | 0  0 | 0  2 | 0  0 | 0  0 | 100  75.0 |
| **Mad**  2016 | no | 2 | 0 | 2 | 0 | 0 | 0 | 0 | 0 | 0 | 0 | 100 |
|  | yes |  | 0 | 2 | 0 | 0 | 0 | 0 | 0 | 0 | 0 | 100 |
| **Mad** | no | 3 | 0 | 3 | 0 | 0 | 0 | 0 | 0 | 0 | 0 | 100 |
| 2017 | yes |  | 0 | 2 | 0 | 0 | 0 | 0 | 1 | 0 | 0 | 66.7 |
| **Blaq**  2016 | no | 9 | 0 | 0 | 9 | 0 | 0 | 0 | 0 | 0 | 0 | 100 |
|  | yes |  | 0 | 0 | 8 | 0 | 0 | 0 | 0 | 1 | 0 | 88.9 |
| **Blaq** | no | 7 | 0 | 0 | 7 | 0 | 0 | 0 | 0 | 0 | 0 | 100 |
| 2017 | yes |  | 2 | 0 | 3 | 0 | 0 | 0 | 2 | 0 | 0 | 42.9 |
| **Cor**  2016 | no | 6 | 0 | 0 | 0 | 6 | 0 | 0 | 0 | 0 | 0 | 100 |
|  | yes |  | 0 | 0 | 0 | 6 | 0 | 0 | 0 | 0 | 0 | 100 |
| **Cor** | no | 9 | 0 | 0 | 0 | 8 | 0 | 0 | 1 | 0 | 0 | 89.9 |
| 2017 | yes |  | 1 | 0 | 0 | 7 | 1 | 0 | 0 | 0 | 0 | 77.8 |
| **Ver**  2016 | no | 2 | 0 | 0 | 0 | 0 | 2 | 0 | 0 | 0 | 0 | 100 |
|  | yes |  | 0 | 0 | 0 | 0 | 2 | 0 | 0 | 0 | 0 | 100 |
| **Ver** | no | 5 | 0 | 0 | 0 | 0 | 5 | 0 | 0 | 0 | 0 | 100 |
| 2017 | yes |  | 0 | 1 | 0 | 2 | 2 | 0 | 0 | 0 | 0 | 40.0 |
| **Gal**  2016 | no | 4 | 0 | 0 | 0 | 0 | 0 | 4 | 0 | 0 | 0 | 100 |
|  | yes |  | 0 | 0 | 0 | 0 | 0 | 4 | 0 | 0 | 0 | 100 |
| **Gal** | no | 22 | 0 | 0 | 0 | 0 | 0 | 22 | 0 | 0 | 0 | 100 |
| 2017 | yes |  | 0 | 0 | 0 | 0 | 0 | 22 | 0 | 0 | 0 | 100 |
| **Carr**  2016 | no | 2 | 0 | 0 | 0 | 0 | 0 | 0 | 2 | 0 | 0 | 100 |
|  | yes |  | 0 | 0 | 0 | 0 | 0 | 0 | 2 | 0 | 0 | 100 |
| **Carr** | no | 7 | 0 | 0 | 0 | 0 | 0 | 0 | 7 | 0 | 0 | 100 |
| 2017 | yes |  | 3 | 0 | 0 | 0 | 0 | 0 | 2 | 1 | 1 | 28.6 |
| **Arb**  2016 | no | 5 | 0 | 0 | 0 | 0 | 0 | 0 | 0 | 5 | 0 | 100 |
|  | yes |  | 3 | 0 | 0 | 0 | 0 | 0 | 0 | 2 | 0 | 40.0 |
| **Arb** | no | 12 | 0 | 0 | 0 | 0 | 0 | 0 | 0 | 12 | 0 | 100 |
| 2017 | yes |  | 0 | 0 | 0 | 0 | 0 | 0 | 0 | 12 | 0 | 100 |
| **Pic**  2016 | no | 5 | 0 | 0 | 0 | 0 | 0 | 0 | 0 | 0 | 5 | 100 |
|  | yes |  | 0 | 0 | 0 | 0 | 0 | 1 | 0 | 0 | 4 | 80.0 |
| **Pic** | no | 6 | 0 | 0 | 0 | 0 | 0 | 0 | 0 | 0 | 6 | 100 |
| 2017 | yes |  | 0 | 0 | 0 | 2 | 0 | 0 | 0 | 0 | 4 | 66.7 |
| **Total**  2016 | no | 42 | 7 | 2 | 9 | 6 | 2 | 4 | 2 | 5 | 5 | 100 |
|  | yes |  | 8 | 2 | 8 | 6 | 2 | 5 | 2 | 5 | 4 | 83.3 |
| **Total** | no | 83 | 12 | 3 | 7 | 8 | 5 | 22 | 8 | 12 | 6 | 98.8 |
| 2017 | yes |  | 15 | 3 | 4 | 11 | 3 | 22 | 7 | 13 | 5 | 75.9 |

Cob - Cobrançosa; Mad - Madural; Blaq - Blanqueta; Cor - Cordovil de Serpa; Ver - Verdeal Alentejana; Gal - Galega Vulgar; Carr - Carrasquenha; Arb - Arbequina; Pic - Picual. CV - Cross validation
